# Multiple Twin Boundaries in Co-Free Li-Rich Mn-Based Cathodes Constructed by Na-Assisted Sol–Gel Synthesis for Enhanced Electrochemical Performance

**DOI:** 10.3390/nano16110674

**Published:** 2026-05-27

**Authors:** Zhihao Jin, Guohua Li, Jiantao Wang, Zhuo Huang

**Affiliations:** 1 China Automotive Battery Research Institute Co., Ltd., Beijing 101407, China; 2 National Power Battery Innovation Center, Grinm Group Corporation Limited, Beijing 100088, China; 3 General Research Institute for Nonferrous Metals, Beijing 100088, China

**Keywords:** cobalt-free Li-rich Mn-based cathode, Na-assisted sol–gel strategy, multiple twin boundaries, crystallographic defect engineering

## Abstract

Cobalt-free Li-rich Mn-based layered oxides are promising cathode materials for next-generation lithium-ion batteries because of their high capacity and reduced reliance on cobalt resources. However, their practical application is still limited by low initial Coulombic efficiency, sluggish reaction kinetics, severe voltage decay, and progressive structural degradation during cycling. In this work, a Na-assisted sol–gel strategy was developed to construct a cobalt-free Li-rich Mn-based cathode with multiple twin boundaries, and the optimized sample with the composition of Li_1.13_Na_0.06_Mn_0.594_Ni_0.219_O_2_ was denoted as SG-TB. Unlike conventional surface coating or elemental doping, this strategy focuses on regulating the bulk crystal framework through crystallographic defect engineering. Structural characterizations indicate that SG-TB contains repeatedly distributed twin-boundary-related interfaces, supporting the presence of multiple twin boundaries within the layered cathode. Benefiting from this structural feature, SG-TB delivers an initial Coulombic efficiency of 96%, an initial discharge capacity of 256 mAh/g, a discharge capacity of 167 mAh/g at 5 C, and a capacity retention of 77% after 200 cycles at 1 C. Further analyses suggest that the multiple twin boundaries help reduce electrochemical polarization, enhance Li^+^ diffusion kinetics, and improve structural retention during cycling. This work demonstrates that Na-assisted multiple twin-boundary engineering is an effective strategy for improving the reaction reversibility and structural stability of cobalt-free Li-rich Mn-based cathodes.

## 1. Introduction

Lithium-ion batteries have become the dominant electrochemical power source for portable electronics, electric vehicles, and increasingly large-scale energy-storage systems because of their high energy density, long service life, and mature manufacturing infrastructure [1,2]. As the demand for electrified transportation continues to rise, cathode materials are required to deliver not only higher capacity but also lower cost and better long-term stability [3,4,5]. In this context, Li-rich Mn-based layered oxides have attracted extensive attention as one of the most promising next-generation cathode families because they can provide high reversible capacity through the combined participation of cationic and anionic redox processes [5,6,7]. Their Mn-rich and potentially cobalt-lean or cobalt-free compositions also make them attractive from the viewpoint of resource sustainability and cost reduction [8]. However, despite these advantages, their practical application remains severely restricted by several intrinsic problems, including low initial Coulombic efficiency, sluggish Li^+^ transport, pronounced voltage decay, and progressive structural deterioration during cycling [9,10,11,12,13]. These problems are closely associated with irreversible oxygen release, transition-metal migration, local cation disorder, and partial reconstruction of the layered framework during high-voltage delithiation/lithiation [10,14,15,16]. Therefore, how to reduce irreversible reactions, improve ion transport, and enhance the stability of the layered framework while preserving the high-capacity advantage has become a key issue for the further development of Li-rich Mn-based cathodes.

To address these issues, various regulation strategies have been explored, including elemental doping, surface coating, and morphology control [17,18,19,20,21]. These strategies can improve certain electrochemical properties by stabilizing surface chemistry, suppressing side reactions, enhancing the reversibility of oxygen redox, or reducing transition-metal migration. However, such approaches mainly rely on external surface protection or chemical composition adjustment, and their ability to achieve coordinated regulation of bulk structural evolution and reaction kinetics remains limited. Compared with conventional doping and coating strategies, crystallographic defect engineering provides an alternative route for tuning the local structure [22]. Without significantly altering the overall chemical composition of the material, this strategy can regulate ion transport, interfacial reactions, and structural evolution at the lattice level, thereby offering new opportunities for synergistically optimizing multiple performance metrics [23,24].

Among various crystallographic defects, twin boundaries are particularly attractive because they are ordered planar defects with crystallographic coherence, rather than random grain boundaries or highly disordered defective regions [25,26]. Such coherent interfaces can alter the local lattice arrangement and may regulate structural evolution during repeated lithiation and delithiation [23,27]. Previous studies have shown that, in classical layered nickel-manganese-cobalt (NMC) cathodes, controlled twin boundaries can mitigate anisotropic lattice changes during high-voltage cycling and act as internal structural support units to improve cycling stability [28]. Twin boundaries induced by non-equilibrium phase transition have also been introduced into spinel lithium manganate systems, significantly improving the electrochemical performance of spinel cathodes [29]. These findings further indicate that twin-boundary engineering has strong potential for regulating ion transport and structural stability in cathode materials.

In addition, ion-exchange synthesis has recently been considered an important route for constructing defects in materials [30]. Ion exchange can regulate alkali-metal-site occupancy, oxygen stacking modes, and local coordination environments, thereby enabling access to metastable structures that are difficult to obtain through conventional high-temperature solid-state synthesis [12,31]. In particular, Li^+^/Na^+^ exchange or Na-assisted local structural rearrangement may induce local lattice mismatch, stacking rearrangement, and alkali-metal-layer disturbance because Na^+^ has a larger ionic radius and different coordination preference than Li^+^ [31,32,33]. It should be noted that the Na-assisted structural rearrangement discussed here is different from conventional Na doping. Conventional Na doping mainly regulates the alkali-metal layer by introducing a certain amount of Na, thereby modifying local structural evolution and improving structural stability [34,35]. In contrast, in Na-assisted structural rearrangement, previous studies have shown that extensive Li^+^/Na^+^ exchange during the Li/Na-containing molten-salt sintering process can effectively introduce defects into materials [32]. This process involves successive phase transitions, intermediate Li/Na mixed states, and topological structural transformation, all of which provide favorable conditions for defect formation in the material [33,36,37].

At present, most twin-boundary design strategies are still mainly focused on classical NMC and spinel oxide systems, and they generally rely on specific solid-state synthesis routes [28,29]. In contrast, studies on constructing twin boundaries in Li-rich Mn-based cathode materials through Na-assisted structural rearrangement remain very limited. Based on this consideration, in this work, a Co-free Li-rich Mn-based layered cathode was synthesized by a sol–gel method. The electrochemical performance of the final products was optimized by regulating the Na content and the Li-rich component ratio, and the Na-assisted structural rearrangement product with the best performance was selected. Compared with the conventional solid-state route, the sol–gel method enables more homogeneous cation mixing at the precursor scale, which is beneficial for obtaining layered oxide materials with more uniform compositional distribution [38,39]. Meanwhile, this method also facilitates sufficient reaction of the Li/Na components during the subsequent heat-treatment process, which is favorable for the formation of multiple twin defects. By adjusting the Na addition amount and the Li-rich component composition, and based on electrochemical performance screening, the optimized sample with high initial Coulombic efficiency and superior rate/cycling performance was denoted as SG-TB. Further characterizations revealed that SG-TB possessed distinct multiple twin-boundary features. Subsequently, X-ray diffraction (XRD), inductively coupled plasma (ICP) analysis, scanning electron microscopy (SEM), transmission electron microscopy (TEM), fast Fourier transform/inverse fast Fourier transform (FFT/IFFT) analysis, electron backscatter diffraction (EBSD), and electrochemical measurements were employed to systematically investigate its crystal structure, composition, morphology, twin-boundary characteristics, and electrochemical behavior. Compared with the Co-free Li-rich Mn-based cathode without obvious twin-boundary features, SG-TB exhibits a higher first-cycle discharge capacity, lower irreversible capacity loss, faster Li^+^ transport kinetics, and more stable cycling performance. This study provides a new perspective for defect-engineering design of Co-free Li-rich Mn-based cathode materials.

## 2. Materials and Methods

### 2.1. Synthesis of the Samples

A series of Co-free Li-rich Mn-based layered oxide cathode materials were synthesized via a sol–gel route. Lithium acetate (LiC_2_H_3_O_2_·2H_2_O, analytical reagent, Shanghai Macklin Biochemical Co., Ltd., Shanghai, China) was used as the lithium source, anhydrous sodium acetate (NaC_2_H_3_O_2_, analytical reagent, Shanghai Macklin Biochemical Co., Ltd., Shanghai, China) as the sodium source, manganese acetate (MnC_4_H_6_O_4_·4H_2_O, analytical reagent, Shanghai Macklin Biochemical Co., Ltd., Shanghai, China) as the manganese source, nickel acetate (NiC_4_H_6_O_4_·4H_2_O, analytical reagent, Shanghai Macklin Biochemical Co., Ltd., Shanghai, China) as the nickel source, citric acid (C_6_H_8_O_7_, analytical reagent, Shanghai Macklin Biochemical Co., Ltd., Shanghai, China) as the chelating agent, and deionized water (H_2_O, prepared in-house) as the solvent. It should be noted that sodium acetate was not introduced to intentionally incorporate Na into the target stoichiometric composition of the final oxide. Instead, it was added as an extra sodium salt during synthesis to regulate precursor reactions and phase-composition evolution during high-temperature calcination. Therefore, the Na present in the final products should be regarded as residual Na, and its actual content was determined according to the subsequent ICP results.

To identify an appropriate compositional window for synthesis, both the Na addition amount and the proportion of the Li-rich component were systematically screened. The target oxide compositions were designed as xLi_2_MnO_3_·(1 − x)LiNi_0.5_Mn_0.5_O_2_, where x = 0.2, 0.3, 0.35, 0.4, and 0.5. After normalization to O_2_, the corresponding nominal compositions were Li_1.091_Ni_0.364_Mn_0.545_O_2_, Li_1.13_Ni_0.304_Mn_0.565_O_2_, Li_1.149_Ni_0.277_Mn_0.574_O_2_, Li_1.167_Ni_0.25_Mn_0.583_O_2_, and Li_1.2_Ni_0.2_Mn_0.6_O_2_, respectively.

First, the effect of Na addition amount was investigated using Li_1.149_Ni_0.277_Mn_0.574_O_2_ as the fixed target composition. The amount of sodium acetate was designed based on the theoretical molar amount of the target oxide product, namely, n(NaC_2_H_3_O_2_)/n(Li_1.149_Ni_0.277_Mn_0.574_O_2_) = 0.1, 0.3, 0.5, 0.7, 0.9, 1.0, 1.2, 1.4, 1.6, and 1.8. When the theoretical yield of the target oxide was set as 1 mol, the corresponding addition amounts of sodium acetate were 0.1, 0.3, 0.5, 0.7, 0.9, 1.0, 1.2, 1.4, 1.6, and 1.8 mol, respectively. These samples were denoted as SG-Na-1 to SG-Na-10 in order of increasing Na addition amount.

In a typical synthesis procedure, the metal salts, lithium source, and sodium acetate were weighed according to the designed stoichiometric ratios and dissolved in deionized water under continuous stirring to form a homogeneous precursor solution. Citric acid was then added as the chelating agent, and the solution was maintained at 80 °C for 12 h to promote complexation and gelation. The obtained wet gel was dried at 160 °C for 24 h to form a xerogel precursor. The dried precursor was first preheated at 400 °C for 5 h to remove residual organic species and then calcined at 850 °C for 12 h in air. After calcination, the resulting powders were washed with deionized water, dried, and sieved to obtain Li-rich Mn-based layered oxide powders synthesized with different Na-assisted conditions.

According to the electrochemical screening results of the SG-Na series, the Na addition ratio with the best overall performance was selected as the fixed condition for subsequent screening of the Li-rich component ratio. Under this optimized Na condition, samples with target compositions of Li_1.091_Ni_0.364_Mn_0.545_O_2_, Li_1.13_Ni_0.304_Mn_0.565_O_2_, Li_1.149_Ni_0.277_Mn_0.574_O_2_, Li_1.167_Ni_0.25_Mn_0.583_O_2_, and Li_1.2_Ni_0.2_Mn_0.6_O_2_ were further synthesized using the same sol–gel procedure. These samples were denoted as SG-x-1 to SG-x-5 in order of increasing Li content in the target composition.

Based on the above screening of Na addition amount and Li-rich component ratio, together with the results of first-cycle charge/discharge profiles, differential capacity (dQ/dV) curves, cycling performance, and discharge median-voltage evolution, the sample with the best overall electrochemical performance was selected for subsequent structural characterization and mechanistic analysis. Further microstructural characterizations, including TEM, FFT, and EBSD, confirmed that this optimized sample possessed distinct multiple twin-boundary features and was therefore denoted as SG-TB. For comparison, a Co-free Li-rich Mn-based layered oxide sample was synthesized by the same sol–gel route without sodium acetate addition, using Li_1.2_Ni_0.2_Mn_0.6_O_2_ as the target stoichiometry, and this sample was denoted as SG-LR.

### 2.2. Materials Characterization

The crystal structures of the as-prepared samples were analyzed by X-ray diffraction (XRD; SmartLab, Rigaku Corporation, Tokyo, Japan) with Cu Kα radiation (λ = 1.5406 Å) over the 2θ range of 10–90° at a scanning rate of 5° min^−1^. Structural refinement and analysis were carried out using GSAS-II software (revision 5782, Argonne National Laboratory, Lemont, IL, USA). The elemental compositions of the samples were determined by inductively coupled plasma optical emission spectroscopy (ICP-OES; 5800, Agilent Technologies, Santa Clara, CA, USA). The particle morphology and surface microstructure were observed by scanning electron microscopy (SEM; S-4800, Hitachi High-Tech Corporation, Tokyo, Japan) equipped with an energy-dispersive X-ray spectroscopy (EDS) detector. High-resolution transmission electron microscopy (HRTEM; Tecnai G2 F20, FEI Company, Hillsboro, OR, USA) was employed to examine the local lattice features, and the corresponding fast Fourier transform (FFT) and inverse fast Fourier transform (IFFT) analyses were conducted to identify crystallographic orientation relationships in representative regions. Electron backscatter diffraction (EBSD; Sigma 300, Carl Zeiss Microscopy GmbH, Jena, Germany) measurements were performed to evaluate the crystallographic orientation distribution and boundary characteristics of the samples. The EBSD data were analyzed using AZtecCrystal software (version 2.1.259, Oxford Instruments, Abingdon, UK).

### 2.3. Electrochemical Measurements

The electrochemical performance was evaluated using CR2032-type coin cells (Shenzhen Kejing Star Technology Co., Ltd., Shenzhen, China). The cathode slurry was prepared by mixing the active material, Super P conductive carbon black (battery grade, Tianjin Youmeng Chemical Technology Co., Ltd., Tianjin, China), and polyvinylidene fluoride (PVDF; analytical reagent, Solvay S.A., Brussels, Belgium) at a weight ratio of 8:1:1, using N-methyl-2-pyrrolidone (NMP; C_5_H_9_NO, analytical reagent, Ningbo Li-Rich Battery Materials Technology Co., Ltd., Ningbo, China) as the solvent. The resulting slurry was uniformly coated onto Al foil (battery grade, Hitachi Metals, Ltd., Tokyo, Japan) and dried at 80 °C. The dried electrodes were then roll-pressed to a compaction density of 2.5 g/cm^3^, punched into disks with a diameter of 14 mm, and further dried under vacuum at 100 °C before cell assembly. The active material loading was controlled at 4 mg/cm^2^. Coin cells were assembled in an Ar-filled glove box using Li metal (battery grade, Tianjin Zhongneng Lithium Industry Co., Ltd., Tianjin, China) as the counter/reference electrode, a polypropylene separator (Celgard 2300, Celgard, LLC, Charlotte, NC, USA), and a high-voltage electrolyte (GLHV-5, Jiangsu Tianci Advanced Materials Co., Ltd., Liyang, China). All electrochemical tests were carried out at 25 ± 2 °C. The specific capacities were calculated based on the mass of active material, and the nominal specific capacity for all subsequent electrochemical tests was set to 200 mAh/g.

Galvanostatic charge/discharge tests, cycling performance tests, and rate capability measurements were performed in the voltage range of 2.0–4.8 V according to the procedures described in the corresponding figure captions. Differential capacity (dQ/dV) curves were derived from the galvanostatic charge/discharge profiles using LAND software (version 7.4, Wuhan LAND Electronic Co., Ltd., Wuhan, China). The first-cycle charge/discharge behavior, long-term cycling stability, and rate performance were compared systematically to evaluate the role of the multi-twin-boundary structure in electrochemical reaction reversibility and kinetic properties.

Galvanostatic intermittent titration technique (GITT) measurements were conducted in the voltage range of 2.0–4.8 V to estimate the apparent Li^+^ diffusion coefficients. During the GITT test, the cells were charged at 0.1 C for 10 min followed by a 1 h relaxation, and the same procedure was repeated until the upper cutoff voltage was reached. The discharge process was then performed with a 10 min current pulse and a 1 h relaxation interval until the lower cutoff voltage was reached. The apparent Li^+^ diffusion coefficients were calculated from the potential response during the intermittent titration process.

## 3. Results and Discussion

To clarify the regulatory effect of sol–gel-derived multiple twin boundaries on the Co-free Li-rich Mn-based cathode, this section discusses the phase structure, microstructure, and electrochemical behavior, with emphasis on the intrinsic mechanism by which multiple twin boundaries enhance Li^+^ transport and structural stability.

### 3.1. Composition Screening and Selection of the SG-TB Cathode

As described in Section 2.1, the optimized Na-assisted Li-rich Mn-based cathode was identified through a two-step screening process. First, the Na addition ratio was varied while fixing the target composition at Li_1.149_Ni_0.277_Mn_0.574_O_2_. Subsequently, under the optimized Na addition condition, the Li-rich component ratio x in xLi_2_MnO_3_·(1 − x)LiNi_0.5_Mn_0.5_O_2_ was further adjusted. The detailed nominal target compositions and Na addition ratios are summarized in Table S1. It should be noted that Na was introduced as an auxiliary sodium salt during synthesis rather than as the Na component in the target stoichiometry of the final oxide.

**Figure 1:**
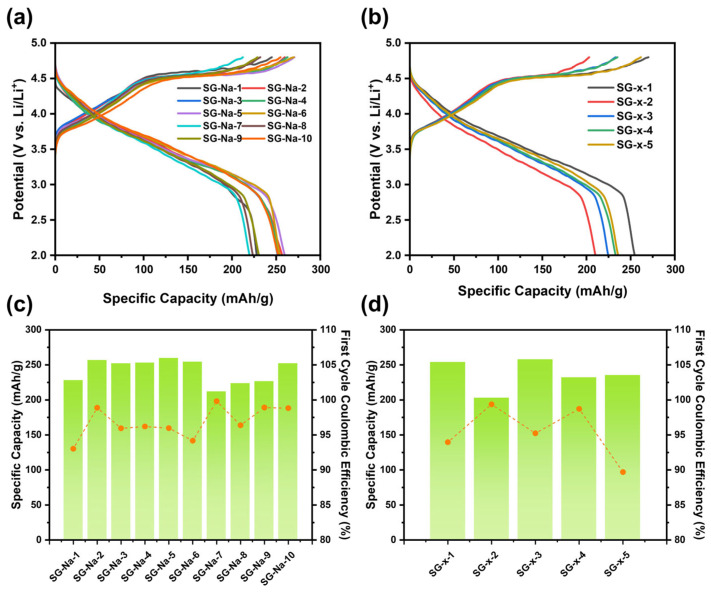
Electrochemical screening of Na-assisted Co-free Li-rich Mn-based cathodes. (**a**) First-cycle charge–discharge curves of SG-Na-1 to SG-Na-10 with different Na addition ratios. (**b**) First-cycle charge–discharge curves of SG-x-1 to SG-x-5 with different Li-rich component ratios. (**c**,**d**) Corresponding first-cycle discharge capacities and initial Coulombic efficiencies of the SG-Na and SG-x series, respectively. The cells were tested in Li half cells within 2.0–4.8 V at 0.1 C. The columns represent discharge capacity, and the dashed lines represent initial Coulombic efficiency.

The effect of Na addition was first examined. As shown in Figure 1a, all samples with different Na addition amounts exhibited typical first-cycle charge–discharge characteristics of Li-rich Mn-based cathodes, including a high-voltage plateau around 4.5 V associated with anionic oxygen redox [40,41]. However, as shown in Figure 1c, obvious differences in first-cycle discharge capacity and initial Coulombic efficiency were observed among the samples with different Na addition amounts. Among them, SG-Na-5 showed better overall performance, delivering a discharge capacity of 256 mAh/g and an initial Coulombic efficiency of 96%. These differences indicate that the Na addition amount affects the initial electrochemical behavior of the materials. Combined with the dQ/dV curves in Figure S1a, shifts in the redox peak positions and changes in peak separation can be identified, suggesting differences in polarization and reaction reversibility among these samples [42]. The evolution of median discharge voltage in Figure S2a further shows that the voltage-retention behavior also depends on the Na addition amount. Based on the above analysis, a Na addition ratio of 0.9 was selected as the final ratio. This part of the screening was mainly used to determine an appropriate Na addition ratio, rather than to directly verify the formation of a twin-boundary structure.

The effect of the Li-rich component ratio x was then investigated. Under the optimized Na addition amount (n(NaC_2_H_3_O_2_)/n(target oxide) = 0.9), the first-cycle charge–discharge curves of the samples with different Li-rich component ratios are shown in Figure 1b. The first-cycle charge–discharge behavior changed markedly with x, indicating that the Li-rich component ratio in the target composition further affected the composition and structure of the final materials. Among these samples, SG-x-3 exhibited the most prominent performance, with the highest discharge capacity and an efficiency above the average value. As shown in Figure 1d, the variation in x led to different capacity outputs and initial reaction reversibility. The dQ/dV curves in Figure S1b further show obvious differences in redox peak intensity and peak separation among the samples with different x values, reflecting differences in reaction polarization. The median discharge voltage results in Figure S2b indicate that x also affects voltage retention during cycling. The XRD patterns of the samples with different x values in Figure S3 show that all samples generally maintained the characteristic layered framework of Li-rich Mn-based oxides, while a small amount of P2-related diffraction features (Na-containing layered structures with prismatic alkali-ion coordination) around 16° was observed in several samples [43]. This indicates that the Li-rich component ratio also influences the phase-composition characteristics of the products, and the detailed phase-composition results will be discussed in the following section.

By comprehensively considering the initial capacity, initial Coulombic efficiency, rate/cycling response, median-voltage retention behavior, and phase composition of the final products, SG-TB, namely SG-x-3, was finally identified as the optimal composition, with a target composition of Li_1.149_Ni_0.277_Mn_0.574_O_2_ and a Na-assisted ratio of n(NaC_2_H_3_O_2_)/n(target oxide) = 0.9. The selection of SG-TB was not based on a single best-performing metric, but on a rational optimization logic involving the balance of multiple indicators: this sample exhibits better initial reaction reversibility, lower polarization, and slower voltage decay during cycling, indicating that this composition can simultaneously balance phase-formation completeness, structural rearrangement capability, and subsequent electrochemical stability. Therefore, SG-TB was selected as the target sample for the following structural characterization and electrochemical investigation.

### 3.2. Crystal Structure, Composition, and Morphology of SG-LR and SG-TB

**Figure 2:**
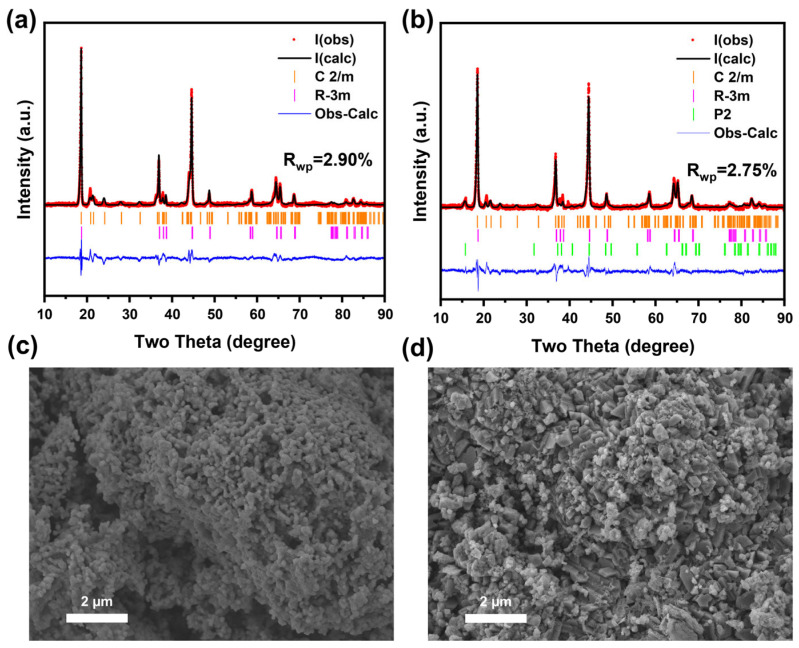
Structural and morphological characterization of SG-LR and SG-TB. (**a**,**b**) XRD patterns and Rietveld refinement results of SG-LR and SG-TB. The dots, solid lines, and bottom lines represent observed data, calculated profiles, and fitting residuals, respectively. (**c**,**d**) SEM images of SG-LR and SG-TB.

After the composition optimization and the identification of the SG-TB formulation, the crystal structure, elemental composition, and microstructural morphology of SG-LR and SG-TB were further systematically characterized to verify that both samples had been successfully synthesized as the targeted Li-rich Mn-based layered cathodes and to provide a basis for the subsequent discussion of the origin of their performance differences. As shown in Figure 2a,b, the XRD patterns of both SG-LR and SG-TB exhibit typical diffraction features of Li-rich layered oxides. The main diffraction peaks can be assigned to the hexagonal R-3m structure commonly observed in Li-rich materials, while the superlattice reflections at 20–25° are associated with the ordered Li_2_MnO_3_ component with a monoclinic C2/m structure, indicating that the obtained products are typical Li-rich Mn-based layered cathode materials. In addition, the clear splitting of the (006)/(102) doublet around 38° and the (108)/(110) doublet around 65° suggests that both samples possess well-defined layered structural ordering [44]. It is worth noting that SG-TB also shows local broadening of the (104) peak around 45°, implying that, while maintaining a stable layered host phase, SG-TB may contain a local structural state distinct from that of SG-LR. This feature provides preliminary structural evidence for the twin-boundary-related microstructure discussed later.

To further understand the crystallographic differences between the two samples, Rietveld refinement was performed on their XRD patterns, as shown in Table 1. The refined lattice parameters of SG-LR and SG-TB show no obvious difference. Due to the residual Na, a small amount of P2-phase is retained in SG-TB. The influence of the P2 phase on material performance is complex and usually tends to deteriorate electrochemical performance, mainly by reducing the capacity reversibility at high voltage [43,45]. The phase refinement results indicate that the content of this phase is very low, approximately 4.7%, suggesting a limited influence on the overall electrochemical performance. In addition, the low P2-phase content indicates that most Na has been removed from the final layered oxide. This process is likely associated with the formation of multiple twin-boundary defects.

The actual elemental ratios of the samples were further verified by ICP analysis, as summarized in Table S2. It can be seen that the elemental contents of both SG-LR and SG-TB are generally consistent with their designed compositions. These results indicate that the sol–gel regulation process did not lead to obvious stoichiometric imbalance, and that the performance differences between the two samples cannot be simply attributed to uncontrolled bulk composition. In other words, both materials remain within a reasonable compositional range, thereby excluding compositional deviation as the primary origin of the electrochemical disparity and making it more convincing to relate the subsequent structural advantages to the regulation of a specific microstructure.

From the morphological perspective, as shown in Figure 2c,d, both SG-LR and SG-TB are composed of micron-sized secondary particles, but they exhibit noticeable differences in particle surface state, edge characteristics, and the connection mode of primary particles. Specifically, SG-LR mainly displays aggregation characteristics of fine particles, with relatively rough particle boundaries. In contrast, SG-TB exhibits block-like connection features, and the connections among its primary particles appear more dispersed. Although the SEM results alone cannot directly confirm the internal defect configuration, these morphological differences at least suggest that SG-TB underwent a crystallization and growth process different from that of SG-LR. In other words, the formation of SG-TB is not merely the result of compositional variation, but is more likely accompanied by a distinct local structural evolution process, which lays the foundation for the subsequent analysis of the twin-boundaries.

Furthermore, the EDS elemental mapping results of SG-TB are presented in Figure S4. Mn, Ni, O, and Na are uniformly distributed across the particles, and no obvious elemental segregation or local enrichment can be observed, indicating good chemical homogeneity of this sample at both particle and microscale levels. Combined with the XRD, ICP, and SEM results, it can be concluded that the intrinsic differences between SG-TB and SG-LR do not mainly originate from the absence of the host phase, abnormal elemental ratios, or obvious compositional segregation, but are more likely related to composition-regulated local crystal-structure evolution. This result also provides an important basis for subsequently correlating the enhanced electrochemical performance with the construction of multiple twin-boundary structures.

### 3.3. Formation and Identification of the Multiple Twin Boundaries

To further determine whether SG-TB developed a characteristic microstructure distinct from that of the conventional sol–gel sample, and to clarify whether this structure possessed definite crystallographic features and statistical significance, TEM and EBSD analyses were carried out for SG-LR and SG-TB. As shown in Figure S5, SG-LR exhibits a relatively homogeneous contrast at low-magnification TEM, and no large-area orientation-transition regions or clear internal interfaces are observed within the particles. The measured interplanar spacing of the (003) plane is 0.482 nm, which is consistent with the typical range of (003) interplanar spacing in Li-rich materials. In contrast, SG-TB shows a more complex local structural contrast under TEM, where multiple regions with abrupt contrast variation and discernible interface contours can be identified inside the particle. This difference suggests that the Li/Na co-regulated sol–gel process does not merely alter the composition or morphology, but also induces deeper crystallographic reorganization within the layered framework.

**Figure 3:**
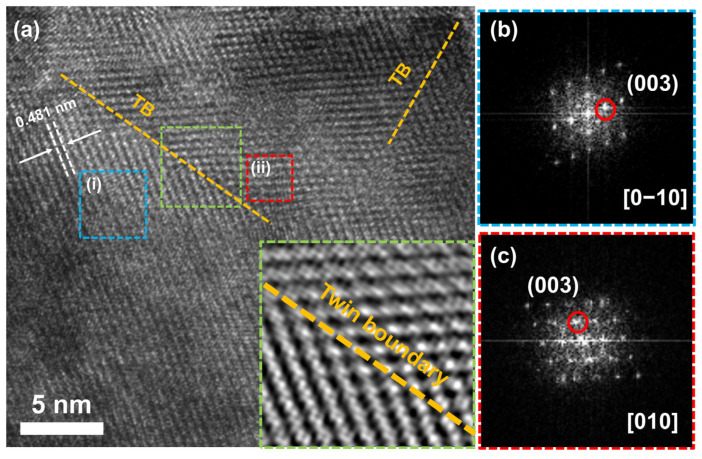
HRTEM analysis of the twin-boundary region in SG-TB. (**a**) HRTEM image of SG-TB showing a representative twin boundary, with the corresponding IFFT image shown in the inset. (**b**,**c**) FFT patterns taken from regions (i) and (ii) on the two sides of the twin boundary, respectively.

As shown in Figure 3a, a distinct internal boundary can be clearly identified in the central region of the particle, across which the lattice fringes on the two sides exhibit a pronounced orientation change, consistent with the feature of a twin boundary (TB). More importantly, such interfaces do not appear as isolated single defects. Instead, they occur repeatedly in adjacent regions and show a tendency toward interconnection within the local domain, indicating that the observed structure should not be described as a single occasional twin boundary, but rather as a multiple twin configuration. Combined with the spatial distribution of several neighboring interfaces in the same particle, SG-TB is inferred to contain repeatedly distributed and interconnected twin boundaries, providing direct microscopic evidence for the formation of multiple twin-boundary structures [33].

To further elevate the above observation from a morphological impression to crystallographic evidence, FFT analysis was conducted on regions i and ii located on the two sides of the interface in Figure 3a. As shown in Figure 3b,c, both regions exhibit clear diffraction spots, indicating that the lattices on both sides remain well ordered. More importantly, the diffraction relationship between the two regions supports a specific orientation correspondence across the interface. In particular, the variation in the diffraction information associated with the (003) planes demonstrates that this interface is not a random grain boundary or arbitrary domain junction, but is crystallographically consistent with a twin relationship. In addition, the IFFT result extracted from the twin region at the lower-right corner of Figure 3a further reveals the interface position and the local continuity of the lattice fringes across the boundary, excluding the possibility that the observed contrast originates from cracks, voids, or amorphous layers. The TEM image of another region shown in Figure S6 also displays multiple twin-distribution features, where the regions on both sides of the twin boundary correspond to the (003) plane and exhibit a mirror relationship of the same plane. Therefore, the combined HRTEM, FFT, and IFFT results confirm the existence of crystallographically well-defined multiple twin boundaries in SG-TB.

**Figure 4:**
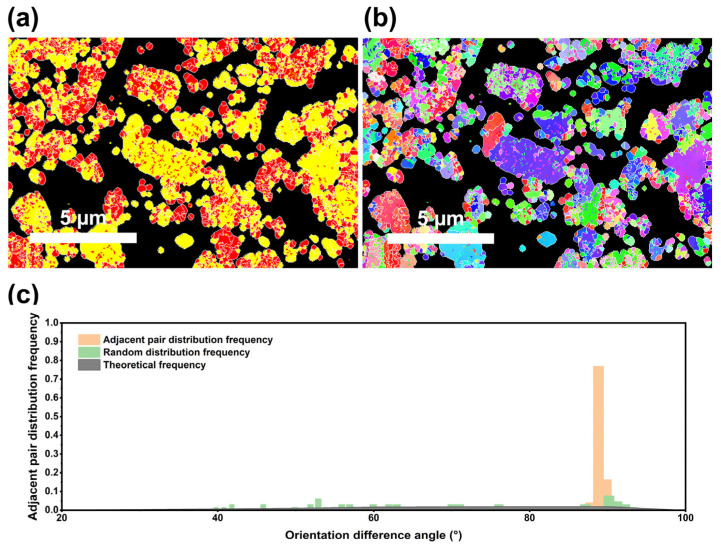
EBSD characterization of SG-TB. (**a**) EBSD phase map, where yellow and red correspond to Li_2_MnO_3_ and LiTMO_2_ (TM = Ni, Mn), respectively. (**b**) Orientation distribution map, with different colors indicating different crystallographic orientations. (**c**) Misorientation-angle distribution, showing preferential orientation relationships related to multiple twin-boundary features.

However, TEM mainly provides local structural evidence. To determine whether such twin-related interfaces are statistically meaningful on a larger scale, EBSD characterization was further performed on SG-TB. As shown in Figure 4a,b, the phase/orientation maps reveal multiple neighboring regions with obvious orientation differences, which agrees well with the local orientation-transition features observed by TEM. More importantly, the misorientation statistics in Figure 4c show a clear enrichment around 90°, indicating that certain orientation relationships are preferentially distributed rather than randomly generated. Although the spatial resolution of EBSD may underestimate the population of extremely fine twin boundaries, it still provides critical large-area statistical support that the twin-related interfaces in SG-TB are not isolated local events, but instead constitute a microstructural feature with measurable spatial distribution and repeated occurrence.

Overall, the TEM, HRTEM, FFT/IFFT, and EBSD results confirm that the Na-assisted sol–gel route can not only induce the formation of twin boundaries, but also further construct multiple twin-boundary structures with statistical significance. This structure is fundamentally different from the relatively homogeneous crystallographic state of SG-LR, represents one of the most important structural characteristics of SG-TB, and provides a key microstructural basis for its subsequent electrochemical behavior.

### 3.4. Electrochemical Advantages of the Multiple Twin Boundaries

After confirming the successful construction of multiple twin boundaries in the SG-TB sample, the next critical question is whether this characteristic microstructure can be translated into measurable electrochemical advantages. To clarify this point, the first-cycle charge/discharge behavior, differential capacity curves, rate capability, and long-term cycling stability of SG-TB and the control SG-LR were systematically compared. The results indicate that multiple twin boundaries not only regulate the local crystallographic features of the cathode, but also significantly improve the first-cycle reaction reversibility, rate response, and structural retention during long-term cycling.

**Figure 5:**
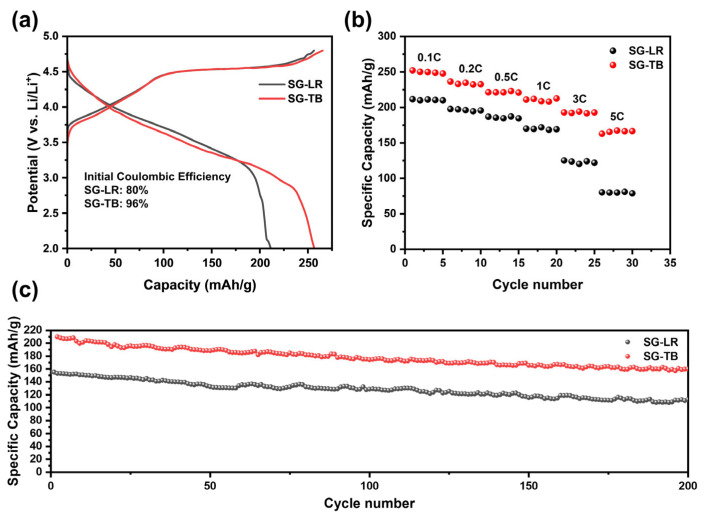
Electrochemical performance of SG-LR and SG-TB. (**a**) First-cycle charge–discharge curves at 0.1 C. (**b**) Rate performance at different current densities. (**c**) Cycling performance at 1 C for 200 cycles. All tests were conducted in Li half cells within 2.0–4.8 V at 25 ± 2 °C, and 1 C was defined as 200 mA/g.

As shown in Figure 5a, SG-TB and SG-LR exhibit evidently different electrochemical behaviors during the initial charge/discharge process. The two samples deliver generally comparable first-charge capacities of around 270 mAh/g. However, SG-TB shows a higher reversible discharge capacity of 256 mAh/g in the first cycle, with an initial Coulombic efficiency of 96%. Compared with SG-LR, which delivers a discharge capacity of 212 mAh/g and an initial Coulombic efficiency of 80%, SG-TB shows a significant improvement. This result indicates that the introduction of multiple twin boundaries effectively suppresses irreversible lithium loss in the initial cycle, enabling the cathode to maintain a higher degree of structural reversibility after the first delithiation process. For Li-rich Mn-based layered cathodes, the irreversible capacity loss in the first cycle is usually associated with local structural instability induced by oxygen activation, interfacial side reactions, and sluggish lithium transport. Therefore, the remarkable increase in the initial Coulombic efficiency of SG-TB suggests that multiple twin boundaries may help alleviate local stress concentration and mitigate structural damage accumulation during the initial activation process, thereby making the first-cycle reaction more reversible.

The rate capability results further demonstrate the kinetic advantage of SG-TB. As shown in Figure 5b, SG-TB delivers higher discharge capacities than SG-LR over the entire tested current range. Under the high-rate condition of 5 C, SG-TB shows a much smaller capacity decay and still maintains a capacity of around 165 mAh/g, whereas SG-LR delivers only about 80 mAh/g at 5 C, showing a significant difference. When the current density returns to a lower value, SG-TB also exhibits a more complete capacity recovery, as shown in Figure S7, indicating that no severe irreversible structural deterioration occurs during fast charge/discharge operation.

The long-term cycling performance further highlights the structural stabilization effect induced by multiple twin boundaries. As shown in Figure 5c, after 200 cycles at 1 C, SG-TB still maintains a higher reversible capacity and better capacity retention than SG-LR, with a capacity retention of approximately 77% for SG-TB and approximately 69% for SG-LR. This result should be interpreted as evidence that multiple twin boundaries enhance structural retention. For Li-rich Mn-based layered cathodes, long-term capacity decay is generally associated with the accumulation of local lattice distortion, aggravated interfacial side reactions, and gradual microstructural degradation.

**Figure 6:**
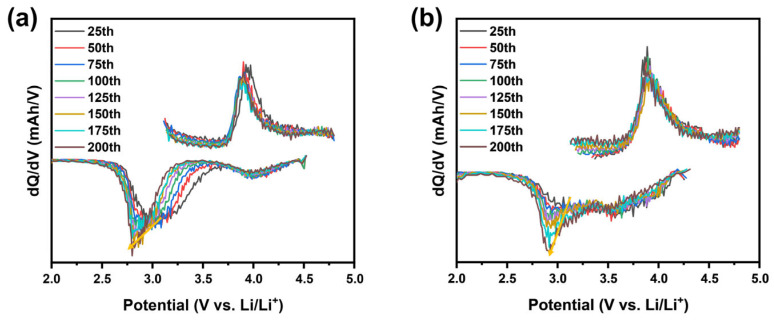
Differential capacity curves of (**a**) SG-TB and (**b**) SG-LR during cycling. The dQ/dV curves were obtained from charge–discharge profiles collected in Li half cells within 2.0–4.8 V at 1 C. The main redox peaks are associated with transition-metal redox and oxygen-related reactions in Li-rich Mn-based layered cathodes.

To further examine the evolution of electrochemical polarization during cycling, the dQ/dV curves of SG-TB and SG-LR from the 25th to the 200th cycle were compared. As shown in Figure 6a, SG-TB maintains relatively well-overlapped oxidation peaks during cycling, and the main oxidation peak remains around 3.8 V with only a small potential shift of approximately 0.1 V. The corresponding reduction peaks also show limited displacement, indicating that SG-TB maintains a relatively stable delithiation/lithiation process during repeated cycling. In contrast, the redox peaks of SG-LR in Figure 6b also show no drastic change, but the increased separation and broadening of the reduction peaks indicate aggravated electrochemical polarization, a change in the capacity-contribution mechanism, and decreased reaction reversibility [46,47]. This result is also consistent with the cycling-performance trend.

The main oxidation peak at approximately 3.8 V can be assigned to transition-metal redox reactions, mainly involving Ni-related redox in the layered framework [41,48]. The reduction peak around 4.0 V is mainly associated with reversible oxygen-related redox [46]. Compared with SG-LR, the smaller shift and better retention of the reduction peak around 4.0 V in SG-TB suggest that multiple twin boundaries help suppress polarization accumulation, reduce oxygen loss, and maintain a more stable structure. In contrast, the evolution of the high-voltage reduction peak in SG-LR corresponds to more pronounced irreversible oxygen loss and spinel-like structural evolution. In addition, as shown in Figure S8, SG-TB still exhibits a more favorable capacity-retention trend after 500 cycles at 1 C, indicating that the stabilizing effect of multiple twin boundaries represents a sustained structural regulation effect.

Combined with the structural analysis in Section 3.3, it can be inferred that the interconnected twin boundaries and orientation-correlated domains provide more favorable microstructural conditions for local stress release and reaction coordination, thereby suppressing polarization accumulation and maintaining continuous electrochemical processes under high-rate operation.

### 3.5. Kinetic Origin of the Enhanced Electrochemical Performance

**Figure 7:**
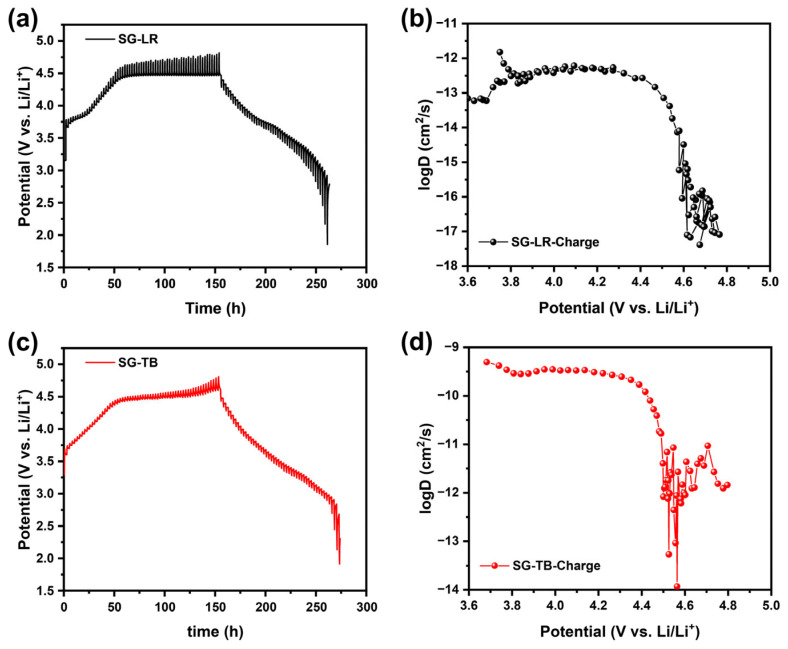
GITT analysis of Li^+^ diffusion kinetics in SG-LR and SG-TB. (**a**,**c**) GITT curves of SG-LR and SG-TB. (**b**,**d**) Apparent Li^+^ diffusion coefficients of SG-LR and SG-TB during charging. The measurements were performed in Li half cells within 2.0–4.8 V using a 0.1 C pulse for 10 min followed by a 1 h relaxation.

The Li^+^ transport behavior of SG-TB was further examined by the galvanostatic intermittent titration technique (GITT) in comparison with SG-LR, in order to reveal the kinetic origin of its enhanced electrochemical performance. As shown in Figure 7a,c, both samples exhibit the typical stepwise voltage response under intermittent current pulses, whereas SG-TB shows a more stable voltage evolution and a smaller transient voltage change during each pulse. This result indicates lower polarization and a faster kinetic response for SG-TB during Li^+^ extraction. The apparent Li^+^ diffusion coefficients calculated from the GITT profiles further confirm the kinetic superiority of SG-TB. As shown in Figure 7b,d, the Li^+^ diffusion coefficients of SG-TB are consistently higher than those of SG-LR during charging. Quantitatively, the Li^+^ diffusion coefficients of SG-TB are approximately distributed in the range of 10^−9.3^ to 10^−13.9^ cm^2^/s, whereas those of SG-LR are approximately in the range of 10^−11.8^ to 10^−17.4^ cm^2^/s. In particular, in the high-voltage region of about 4.55–4.75 V, the diffusion coefficient of SG-LR rapidly decreases to the 10^−16^–10^−17^ cm^2^/s level, while SG-TB still remains mainly in the 10^−11^–10^−13^ cm^2^/s range. This result indicates that SG-TB can maintain much faster Li^+^ transport kinetics even under deep delithiation conditions. In addition, the diffusion-coefficient evolution of SG-TB is smoother over the whole voltage range, suggesting a more homogeneous electrochemical reaction process and fewer local kinetic bottlenecks. The Li^+^ diffusion coefficients during discharge are shown in Figure S9, and their overall trend is consistent with that observed during charge, further demonstrating that SG-TB maintains superior reaction kinetics throughout the entire delithiation/lithiation process.

This kinetic enhancement can be reasonably correlated with the multiple twin-boundary structure constructed in SG-TB. On the one hand, multiple twin boundaries may provide more abundant local diffusion pathways for Li^+^, thereby shortening the effective migration distance and reducing the transport resistance between adjacent domains [49]. On the other hand, multiple twin boundaries may help alleviate local lattice-stress accumulation during repeated Li^+^ extraction/insertion, thereby reducing structural hindrance to ion transport [50]. As a result, SG-TB exhibits not only faster Li^+^ diffusion but also better reaction uniformity. This result is highly consistent with the lower polarization, improved rate capability, and enhanced cycling stability discussed above, further indicating that the electrochemical advantages of SG-TB essentially originate from the structural–kinetic benefits introduced by multiple twin boundaries.

## 4. Conclusions

In summary, a Co-free Li-rich Mn-based layered cathode with multiple twin boundaries, denoted as SG-TB, was successfully developed via a Na-assisted sol–gel strategy. The results indicate that this feature is not merely local defect accumulation, but an effective microstructural unit capable of regulating Li^+^ transport and structural evolution. Benefiting from the introduction of multiple twin boundaries, SG-TB preserves the target layered framework and compositional uniformity while markedly reducing the initial irreversible capacity loss, leading to an increase in the initial Coulombic efficiency from 80% to 96%, together with improved rate capability and cycling stability. Further analysis suggests that multiple twin boundaries help suppress electrochemical polarization and facilitate Li^+^ migration, thereby enhancing reaction reversibility and structural robustness during repeated lithiation/delithiation processes. This work demonstrates that multiple-twin engineering offers a promising structural design strategy for simultaneously achieving high initial efficiency, high rate capability, and high stability in Co-free Li-rich Mn-based cathodes, and provides useful insight into defect regulation of advanced Li-rich cathode materials.

## Figures and Tables

**Table 1:** Crystallographic data were obtained from the Rietveld refinement for SG-LR and SG-TB.

Sample	Phase	Phase Fraction (%)	a (Å)	b (Å)	c (Å)	V (Å^3^)	R_p_ (%)	Rwp (%)
SG-LR	C2/m	57.4	5.007	8.540	5.055	204.263	2.12	2.9
	R-3m	42.6	2.851	2.851	14.250	100.342		
SG-TB	C2/m	53.5	4.961	8.645	5.113	206.112	1.95	2.75
	R-3m	41.8	2.858	2.858	14.297	101.193		
	P2	4.7	2.792	2.792	11.316	76.400		

## Data Availability

The data that support the findings of this study are available from the corresponding author upon reasonable request.

## Supplementary Materials

The following supporting information can be downloaded at: https://www.mdpi.com/article/10.3390/nano16110674/s1, Figure S1: Differential capacity curves of the composition-screening samples; Figure S2: Discharge median-voltage evolution of the composition-screening samples during cycling; Figure S3: XRD patterns of SG-x-1 to SG-x-5 with different Li-rich component ratios; Figure S4: EDS elemental mapping results of the SG-TB sample; Figure S5: TEM characterization of SG-LR; Figure S6: Additional TEM characterization of twin-boundary features in SG-TB; Figure S7: Rate recovery performance of SG-LR and SG-TB; Figure S8: Long-term cycling performance of SG-LR and SG-TB at 1 C for 500 cycles; Figure S9: Apparent Li^+^ diffusion coefficients of SG-LR and SG-TB during discharge calculated from GITT measurements; Table S1: Nominal compositions and NaC_2_H_3_O_2_ addition ratios of the composition-screening samples; Table S2: ICP-derived elemental compositions of SG-LR and SG-TB.
