# Supplementary material for: Multiple Twin Boundaries in Co-Free Li-Rich Mn-Based Cathodes Constructed by Na-Assisted Sol–Gel Synthesis for Enhanced Electrochemical Performance
Source: Nanomaterials (Basel). 2026 May 27;16(11):674. doi: 10.3390/nano16110674 (PMC13258553; doi:10.3390/nano16110674)
Supplement: Supplementary file 1 [file nanomaterials-16-00674-s001.zip › nanomaterials-4297256-supplementary.pdf]

nanomaterials

Supplementary Materials

Multiple Twin Boundaries in Co-Free Li-Rich Mn-Based Cathodes Constructed by Na-Assisted Sol–Gel Synthesis for Enhanced Electrochemical Performance

Zhihao Jin [1,2,3], Guohua Li [1,2,3], Jiantao Wang [1,2,3,*] and Zhuo Huang [1,2,3,*]

1 China Automotive Battery Research Institute Co., Ltd., Beijing 101407, China
2 National Power Battery Innovation Center, Grinm Group Corporation Limited, Beijing 100088, China
3 General Research Institute for Nonferrous Metals, Beijing 100088, China
* Correspondence: wangjt@glabat.com (J.W.); huangzh@grinm.com (Z.H.)

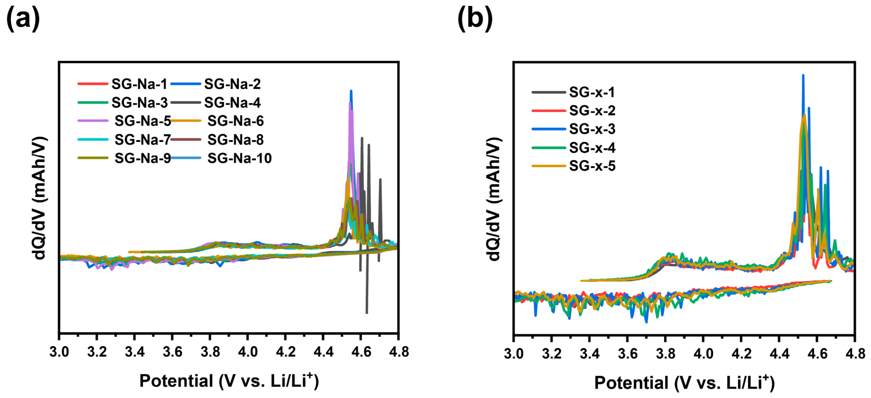

Figure S1. Differential capacity curves of the composition-screening samples. (a) dQ/dV curves of SG-Na-1 to SG-Na-10 with different NaAc addition ratios. (b) dQ/dV curves of SG-x-1 to SG-x-5 with different Li-rich component ratios. The curves were derived from galvanostatic charge-discharge profiles collected in Li half cells within 2.0-4.8 V at 1 C.

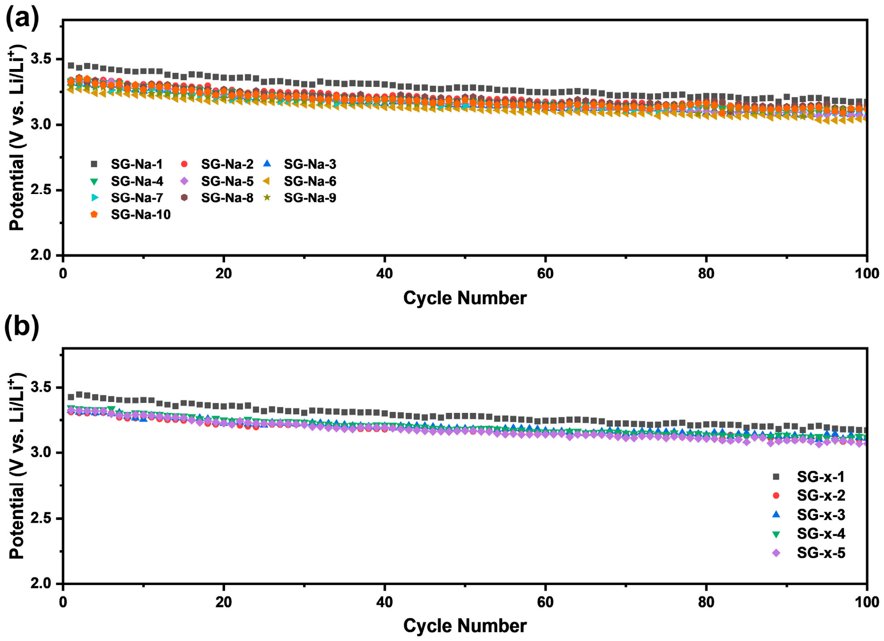

Figure S2. Discharge median-voltage evolution of the composition-screening samples during cycling. (a) Samples with different NaAc addition ratios. (b) Samples with different Li-rich component ratios. The median voltage was calculated from the corresponding discharge profiles to evaluate voltage stability during cycling.The curves were derived from galvanostatic charge-discharge profiles collected in Li half cells within 2.0-4.8 V at 1 C.

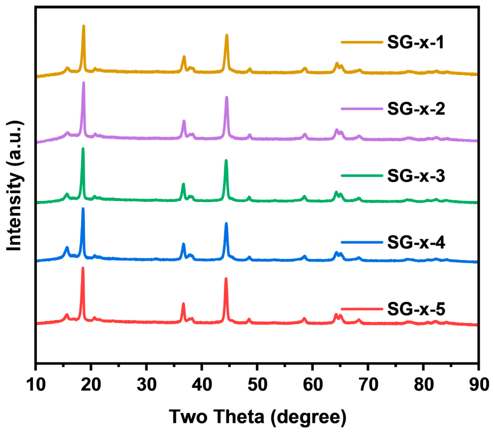

Figure S3. XRD patterns of SG-x-1 to SG-x-5 with different Li-rich component ratios. The samples were prepared under the optimized NaAc-assisted sol-gel condition. All samples show the characteristic diffraction features of Li-rich Mn-based layered oxides.

https://doi.org/10.3390/nano16110674

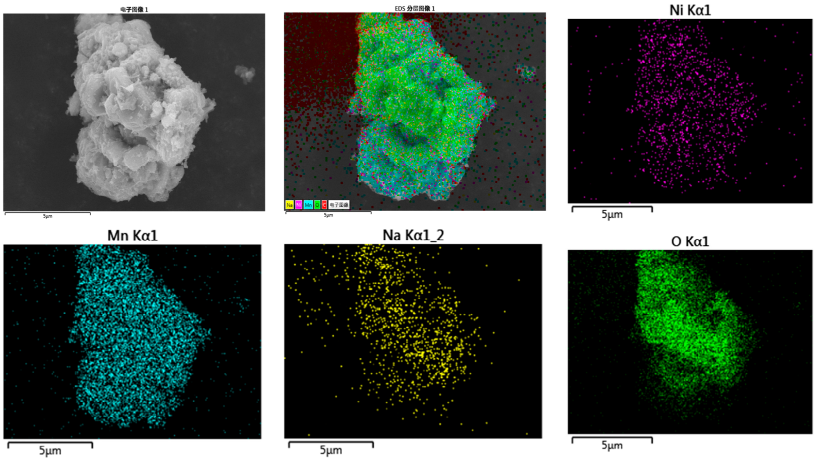

Figure S4. EDS elemental mapping results of the SG-TB sample. The morphology image of the selected region together with the elemental maps of Mn, Ni, O, and Na shows a generally uniform elemental distribution throughout the particles, without obvious local enrichment or elemental segregation.

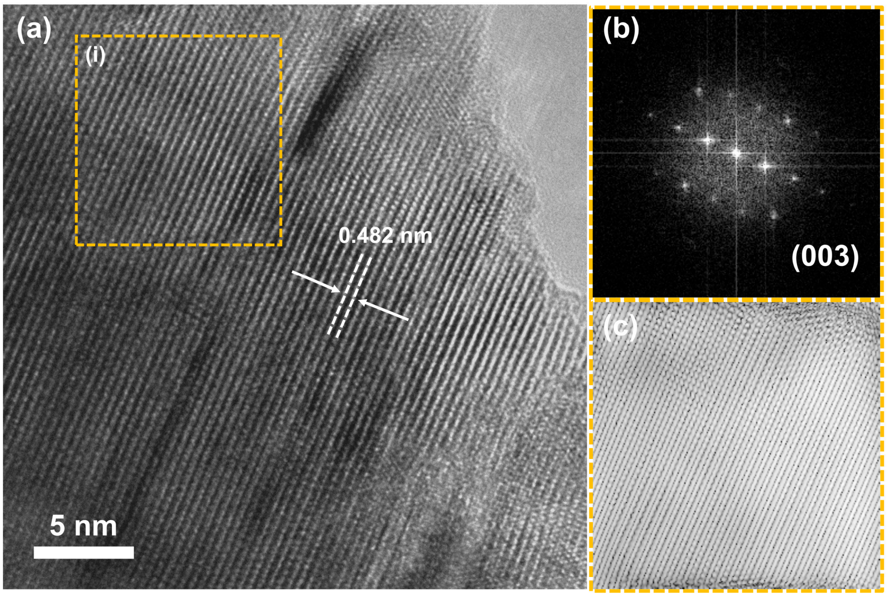

Figure S5. TEM characterization of SG-LR. (a) HRTEM image of a representative region in SG-LR. The measured interplanar spacing is 0.482 nm, corresponding to the (003) plane of the Li-rich layered oxide. (b) FFT pattern taken from the i region marked in (a). (c) Corresponding IFFT image of the i region. No obvious twin-boundary-related interface is observed in this region.

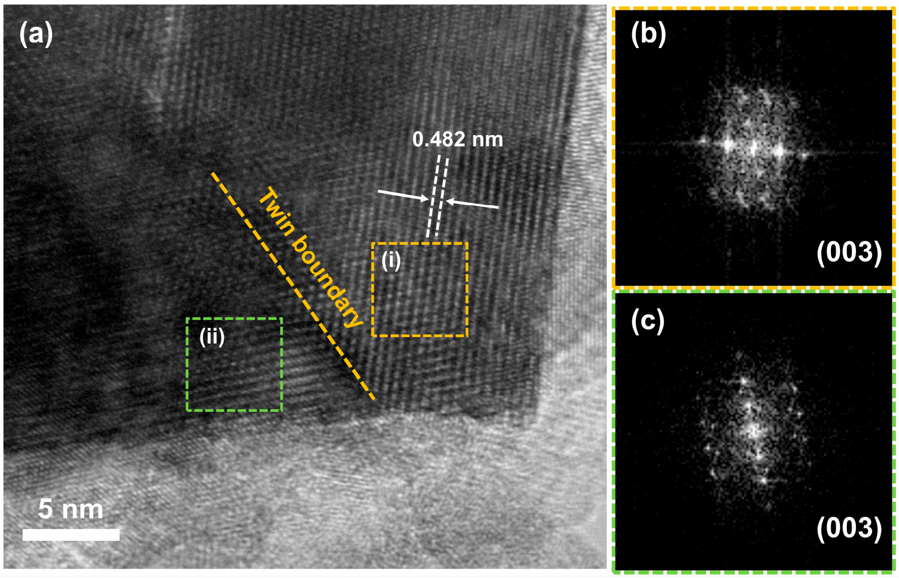

Figure S6. Additional TEM characterization of twin-boundary features in SG-TB. (a) HRTEM image of a representative SG-TB region showing a clear twin boundary. The measured interplanar spacing is 0.482 nm, corresponding to the (003) plane of the Li-rich layered oxide. (b,c) FFT patterns taken from regions i and ii in (a), respectively, showing the 003-related lattice features on the two sides of the twin boundary.

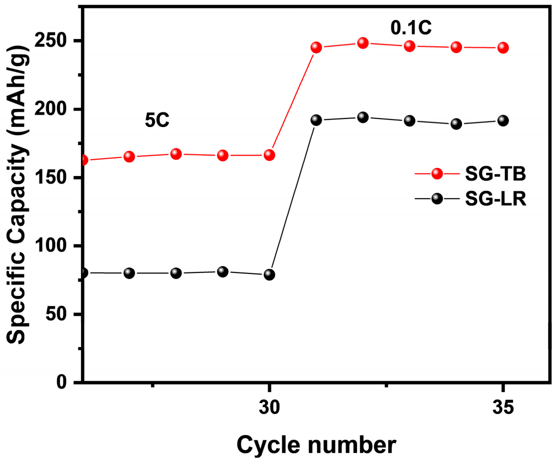

Figure S7. Rate recovery performance of SG-LR and SG-TB. The cells were tested at different current densities and then returned to 0.1 C to evaluate capacity recovery. SG-TB shows better capacity retention and recovery behavior after high-rate cycling.

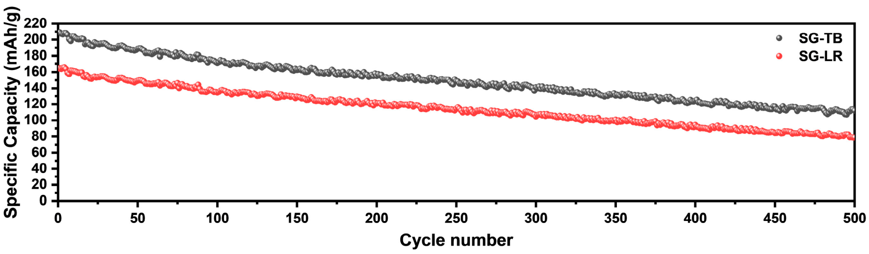

Figure S8. Long-term cycling performance of SG-LR and SG-TB at 1 C for 500 cycles. The cells were tested in Li half cells within 2.0–4.8 V at 25 ± 2°C, and 1 C was defined as 200 mA/g.

https://doi.org/10.3390/nano16110674

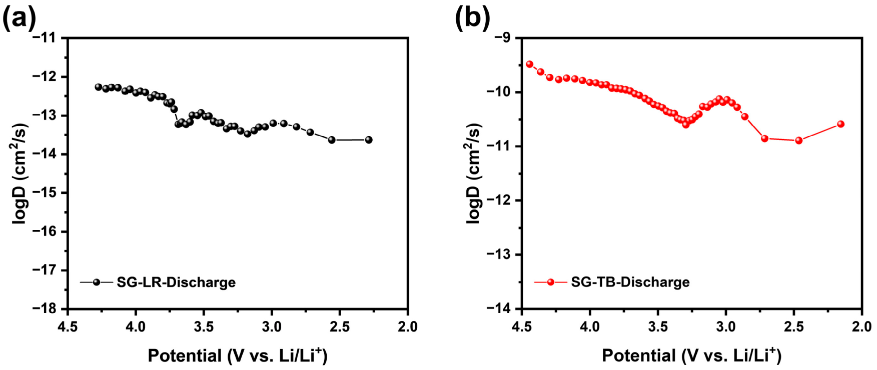

Figure S9. Apparent Li^+ diffusion coefficients of SG-LR and SG-TB during discharge calculated from GITT measurements. The results were obtained from cells within 2.0–4.8 V using a 0.1 C current pulse for 10 min followed by a 1 h relaxation period. (a) apparent Li^+ diffusion coefficients of SG-LR during discharge; (b) apparent Li^+ diffusion coefficients of SG-TB during discharge.

Table S1. Nominal compositions and $NaC_2H_3O_2$ addition ratios of the composition-screening samples.

Sample	Nominal target composition	Normalized formula	n($NaC_2H_3O_2$)/n(target oxide)
SG-Na-1	$0.35Li_2MnO_3·0.65LiNi_{0.5}Mn_{0.5}O_2$	$Li_{1.149}Ni_{0.277}Mn_{0.574}O_2$	0.1
SG-Na-2	$0.35Li_2MnO_3·0.65LiNi_{0.5}Mn_{0.5}O_2$	$Li_{1.149}Ni_{0.277}Mn_{0.574}O_2$	0.3
SG-Na-3	$0.35Li_2MnO_3·0.65LiNi_{0.5}Mn_{0.5}O_2$	$Li_{1.149}Ni_{0.277}Mn_{0.574}O_2$	0.5
SG-Na-4	$0.35Li_2MnO_3·0.65LiNi_{0.5}Mn_{0.5}O_2$	$Li_{1.149}Ni_{0.277}Mn_{0.574}O_2$	0.7
SG-Na-5	$0.35Li_2MnO_3·0.65LiNi_{0.5}Mn_{0.5}O_2$	$Li_{1.149}Ni_{0.277}Mn_{0.574}O_2$	0.9
SG-Na-6	$0.35Li_2MnO_3·0.65LiNi_{0.5}Mn_{0.5}O_2$	$Li_{1.149}Ni_{0.277}Mn_{0.574}O_2$	1.0
SG-Na-7	$0.35Li_2MnO_3·0.65LiNi_{0.5}Mn_{0.5}O_2$	$Li_{1.149}Ni_{0.277}Mn_{0.574}O_2$	1.2
SG-Na-8	$0.35Li_2MnO_3·0.65LiNi_{0.5}Mn_{0.5}O_2$	$Li_{1.149}Ni_{0.277}Mn_{0.574}O_2$	1.4
SG-Na-9	$0.35Li_2MnO_3·0.65LiNi_{0.5}Mn_{0.5}O_2$	$Li_{1.149}Ni_{0.277}Mn_{0.574}O_2$	1.6
SG-Na-10	$0.35Li_2MnO_3·0.65LiNi_{0.5}Mn_{0.5}O_2$	$Li_{1.149}Ni_{0.277}Mn_{0.574}O_2$	1.8
SG-x-1	$0.2Li_2MnO_3·0.8LiNi_{0.5}Mn_{0.5}O_2$	$Li_{1.09}1Ni_{0.364}Mn_{0.545}O_2$	0.9
SG-x-2	$0.3Li_2MnO_3·0.7LiNi_{0.5}Mn_{0.5}O_2$	$Li_{1.13}Ni_{0.304}Mn_{0.565}O_2$	0.9
SG-x-3	$0.35Li_2MnO_3·0.65LiNi_{0.5}Mn_{0.5}O_2$	$Li_{1.149}Ni_{0.27}7Mn_{0.574}O_2$	0.9
SG-x-4	$0.4Li_2MnO_3·0.6LiNi_{0.5}Mn_{0.5}O_2$	$Li_{1.167}Ni_{0.25}Mn_{0.583}O_2$	0.9
SG-x-5	$0.5Li_2MnO_3·0.5LiNi_{0.5}Mn_{0.5}O_2$	$Li_{1.2}Ni_{0.2}Mn_{0.6}O_2$	0.9
SG-TB	$0.35Li_2MnO_3·0.65LiNi_{0.5}Mn_{0.5}O_2$	$Li_{1.149}Ni_{0.27}7Mn_{0.574}O_2$	0.9
SG-LR	$0.5Li_2MnO_3·0.5LiNi_{0.5}Mn_{0.5}O_2$	$Li_{1.2}Ni_{0.2}Mn_{0.6}O_2$	0.0

Table S2. ICP-derived elemental compositions of SG-LR and SG-TB.

Sample	Designed composition	ICP results
SG-LR	$Li_{1.2}Mn_{0.6}Ni_{0.2}O_2$	$Li_{1.194}Mn_{0.595}Ni_{0.213}O_2$
SG-TB	$Li_{1.149}Ni_{0.27}7Mn_{0.574}O_2$	$Li_{1.13}Na_{0.06}Mn_{0.594}Ni_{0.219}O_2$

https://doi.org/10.3390/nano16110674
